# Temporal sensitivity and perceived timing in unimodal and crossmodal isochronous sequences

**DOI:** 10.1007/s00221-026-07371-1

**Published:** 2026-08-06

**Authors:** Min Susan Li, Massimiliano Di Luca

**Affiliations:** 1 Present Address: School of Psychology, University of Birmingham, Birmingham, B15 2TT UK; 2 School of Computer Science, University of Birmingham, Birmingham, B15 2TT UK

**Keywords:** Crossmodal timing, Isochrony, Modality expectation, Temporal sensitivity, Perceptual timing

## Abstract

Temporal judgments depend on sequence regularity and on the sensory modality in which events occur. We asked whether modality-specific sequence history and across-trial modality context make distinct contributions to temporal sensitivity and perceived timing in isochrony judgments. Participants judged whether the sixth event in a six-item sequence occurred earlier or later than expected. The first five events were auditory or visual; the final event either matched this modality or switched modality. The four sequence types were presented either in separate blocks or randomly interleaved across trials; this manipulation is referred to as presentation mode. Temporal sensitivity, indexed by the just noticeable difference (JND), was better for auditory than for visual sequences and better for modality-consistent than for modality-inconsistent sequences. Effects involving presentation mode were treated as descriptive because this factor was between participants and confounded with hardware settings. Perceived timing, indexed by the point of subjective equality (PSE), showed large shifts for modality-switch endings in the interleaved group. The results support a dissociation between sequence-related effects on temporal sensitivity and context-dependent biases in perceived timing.

## Introduction

Isochronous sequences are sequences in which successive events occur at equal inter-onset intervals, where the inter-onset interval is the time from the onset of one event to the onset of the next. Such sequences provide a useful way to study how observers form expectations about when a future event should occur. In the present task, participants judged whether the final event in a six-event sequence occurred earlier or later than the time predicted by the preceding regular rhythm. We therefore distinguish temporal sensitivity, measured by the just noticeable difference (JND), from perceived timing, measured by the point of subjective equality (PSE). The JND indexes how precisely participants discriminated early from late final events, whereas the PSE indexes the physical deviation from exact isochrony (anisochrony) at which the final event was equally likely to be judged early or late.

Several theoretical accounts predict that regular sequences can support temporal prediction, but they differ in mechanism and in the aspect of temporal judgement they emphasise. Clock-based accounts describe timing in terms of accumulated pulses or internal temporal representations (Creelman 1962; Treisman 1963; Povel 1981; Wearden 1992). Multiple-look and perceptual-averaging accounts propose that repeated interval samples can reduce uncertainty about the expected time of a subsequent event (Drake and Botte 1993; Schulze 1989). Dynamic Attending Theory and related entrainment accounts propose that rhythmic context can guide attention to expected moments in time (Jones and Boltz 1989; McAuley and Jones 2003). These accounts share the general prediction that temporal regularity can support better discrimination, but they do not require the same interpretation for all forms of temporal judgement. For example, changes in perceived duration and changes in precision can dissociate across tasks and stimulus conditions.

Temporal judgments also depend on sensory modality Di Stefano & Spence (2025). Auditory signals generally support more precise temporal discrimination than visual signals (Repp and Penel 2002; Stauffer et al. 2012; McAuley and Henry 2010; Fornaciai et al. 2018). Auditory and visual markers can also differ in their effective perceptual latencies. They can also shift a decisional reference point, defined here as the subjective criterion used to decide whether the final event occurred before or after the expected isochronous time. Such latency or criterion differences can produce systematic biases in perceived timing and perceived duration for intervals bounded by markers from different sensory modalities (crossmodal intervals; Hirsh and Sherrick 1961; Sternberg and Knoll 1973; Grondin et al. 1996; Mayer et al. 2014).

Recent context can also influence perceived audiovisual timing, although the present study should be distinguished from classical temporal recalibration paradigms. In temporal recalibration studies, repeated exposure to audiovisual stimuli with a fixed temporal offset can shift the PSE for simultaneity or temporal order judgments (Spence and Squire 2003; Di Luca et al. 2009; Harrar and Harris 2008; Spence and Parise 2010; Machulla et al. 2012). The present study did not use repeated bimodal adaptor pairs with fixed offsets. Instead, it asked whether knowledge about the likely modality pattern across trials changes the subjective alignment assigned to the final event of a sequence.

Taken together, these literatures motivate a more specific question. In an isochronous sequence that can contain a switch between sensory modalities (a crossmodal sequence), the observer can use both the temporal template established within the current sequence and the broader context established by the presentation schedule. Previous work on crossmodal intervals suggests that marker modality can affect both precision and bias (Grondin and Rousseau 1991; Grondin and McAuley 2009), and work on rhythmic expectation suggests that temporal asynchrony and expectation can alter perceived duration or timing in related paradigms (McAuley and Jones 2003; Wehrman et al. 2018). Classic variable-foreperiod work also shows that temporal preparation can depend on the timing context established by preceding intervals or trials (Woodrow 1914). This raises the possibility that modality-specific sequence history primarily affects temporal sensitivity, whereas across-trial modality context primarily affects perceived timing.

We tested this possibility using six-event sequences. The first five events were isochronous and were always presented in a single modality (unimodal), auditory or visual. The sixth event either matched that modality (a consistent ending) or switched to the other modality (an inconsistent or crossmodal ending), and it could occur earlier or later than the expected isochronous time. This design yielded four sequence types: Audio-Consistent, Audio-Inconsistent, Visual-Consistent, and Visual-Inconsistent. Presentation mode refers to whether these four sequence types were presented in separate blocks or randomly interleaved across trials. Blocking provided across-trial information about the likely modality pattern, whereas interleaving reduced that information.

Two predictions guided the study. First, if temporal sensitivity depends mainly on the modality pattern established within a sequence, then switching modality at the final event should reduce sensitivity. Second, if perceived timing is influenced by across-trial knowledge about the likely modality sequence, then PSE deviations should be most evident when sequence types are randomly interleaved, particularly when the final event switched modality. Because presentation mode was manipulated between participants and was confounded with hardware settings, evidence involving this factor is interpreted descriptively rather than as an isolated effect of presentation schedule.

## Methods

### Participants

Thirty-six undergraduate students (11 male; mean age = 20.3 years, SD = 2.2) participated in the experiment. Eighteen participants completed the blocked version and 18 completed the interleaved version. The sample size was determined by the recruitment constraints of the original student-participant study and by the repeated-measures design, in which each participant completed multiple trials for each sequence type. The design therefore provides stronger evidence for within-participant effects of sequence modality (whether the first five events were auditory or visual) and consistency (whether the final event matched or switched modality) than for between-participant effects of presentation mode. Evidence involving presentation mode is consequently treated as descriptive and preliminary, especially because the two groups were tested with different hardware settings. Participants reported normal or corrected-to-normal vision and normal hearing, were naive to the purpose of the study, and received either course credit or payment. The study was approved by the Science, Technology, Engineering and Mathematics Ethical Review Committee of the University of Birmingham (ERN_11-1241) and complied with the Declaration of Helsinki. Participants provided informed consent before taking part.

## Design and stimuli

**Fig. 1:**
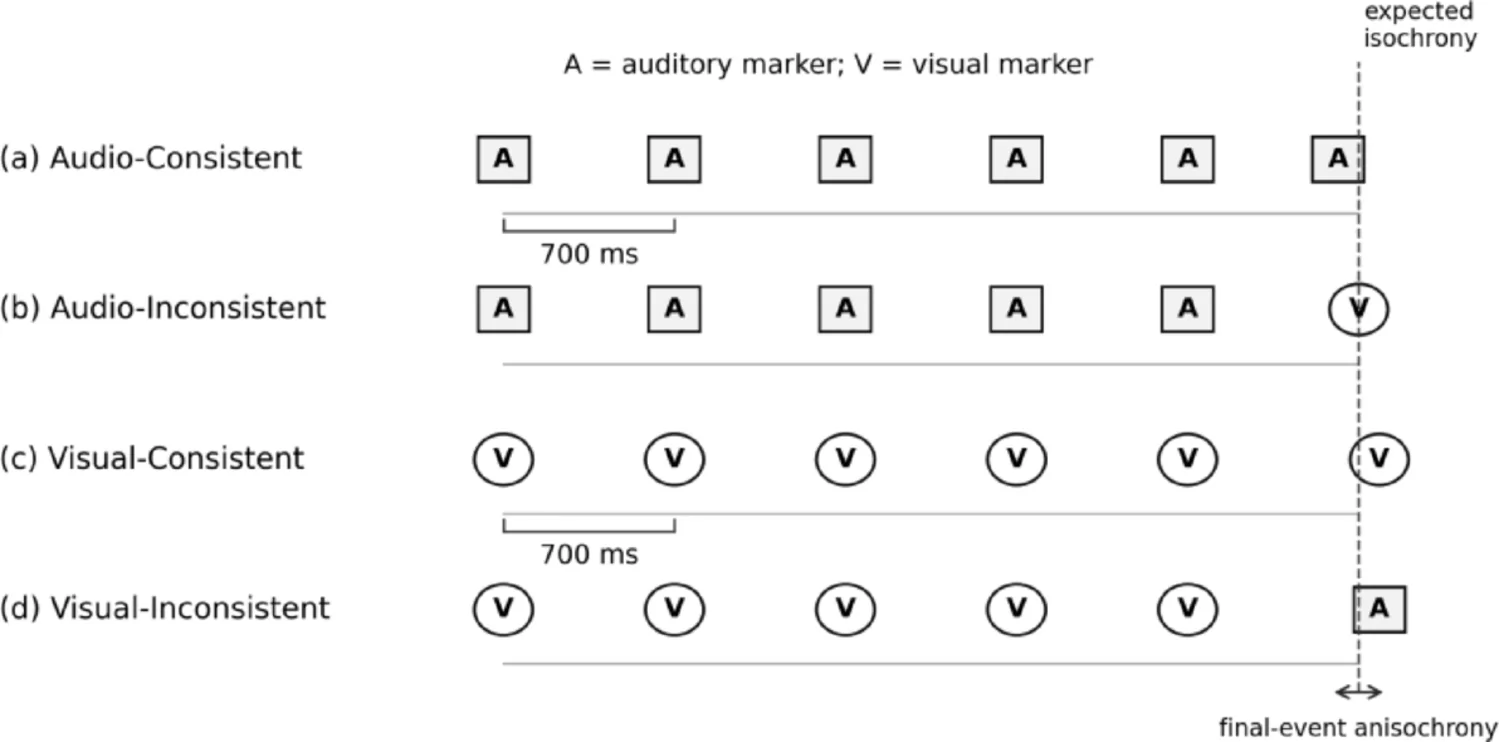
Experimental sequence types. The first five events were auditory or visual and separated by 700 ms; the final event either matched or switched modality and occurred at one of seven anisochronies relative to expected isochrony

Each trial consisted of six events. The first five were separated by an isochronous inter-onset interval of 700 ms. The sixth event could occur at the expected isochronous time or be shifted by one of seven stimulus-onset asynchronies (SOAs), defined here as the onset shift of the final event relative to the expected isochronous time (− 200, − 100, − 50, 0, + 50, +100, + 200 ms). These values were chosen to sample signed early and late deviations symmetrically around nominal isochrony, providing a balance between fine sampling near the expected PSE and broader coverage for estimating JNDs across conditions and participants. The first five events were always unimodal, either all auditory or all visual. The sixth event either matched this modality (consistent) or switched to the other modality (inconsistent), yielding four sequence types: Audio-Consistent, Audio-Inconsistent, Visual-Consistent, and Visual-Inconsistent (Fig. 1). Each SOA was repeated 12 times per sequence type, for a total of 336 experimental trials per participant. Sequence types were presented either in separate blocks or randomly interleaved. In the blocked version, each sequence type was presented in a separate block, and the order of the four blocks was randomised across participants. Presentation mode was manipulated between participants.

## Apparatus and procedure

The stimuli were produced by the two channels of a soundcard, and the experiment was controlled in MATLAB R2016b with Psychophysics Toolbox (Brainard 1997; Pelli 1997; Kleiner et al. 2007) on a Windows 7 PC. Auditory markers were 1000 Hz tones presented through a speaker. Visual markers were brief LED flashes generated from a 100-Hz waveform on the visual channel. The stimulus device was positioned approximately 50 cm in front of the participant in a dark testing cubicle. All stimuli were 20 ms long with a 1-ms on-ramp and off-ramp. Participants indicated by key press whether the sixth event occurred earlier or later than expected from the rhythm established by the preceding five events. Before the experimental trials, participants completed 48 practice trials.

The blocked and interleaved groups were run with different hardware settings for the visual and auditory markers. The blocked group used a yellow LED (luminance 62.2 cd/m2) and an auditory intensity of 84.14 dB, whereas the interleaved group used a red LED (luminance 91.9 cd/m2) and an auditory intensity of 60.9 dB. Because these hardware settings were confounded with presentation group, they cannot be included as independent covariates in the statistical model. PSEs are therefore interpreted as subjective alignment biases of the final event relative to the preceding sequence, rather than as direct measurements of sensory latency.

### Data analysis

For each participant and condition, psychometric data, defined as the proportion of late responses at each SOA, were summarised with the non-parametric Spearman-Karber estimator after monotonic correction. The monotonic correction constrained the response function to increase with later final-event timing. This estimator was used because it provides PSE and JND estimates without assuming a specific parametric psychometric-function shape (Spearman 1908; Kärber 1931; Bausenhart et al. 2018; Ulrich and Miller 2004), which is useful when some conditions produce shallow functions near the edge of the tested SOA range. PSE was estimated from the first moment of the resulting psychometric function and JND from the associated threshold estimate, with lower JND values indicating better sensitivity.

JND and PSE values were analysed with mixed analyses of variance (ANOVAs) including presentation mode (blocked vs. interleaved) as a between-participants factor and sequence modality (auditory vs. visual first five events) and consistency (consistent vs. inconsistent final event) as within-participants factors. Effects involving presentation mode are reported as descriptive checks because this grouping factor was confounded with hardware settings. Interpreted follow-up tests therefore focus on within-participant contrasts among sequence types or on one-sample deviations from isochrony within a presentation group. Partial η squared (η_p_^2^) is reported for all ANOVA effects. Error bars in the figures represent +/− 1 standard error of the mean (SEM) across participants. Because the tested SOA range was limited to − 200 to + 200 ms, large JND values in inconsistent conditions should be interpreted as relative sensitivity indices rather than precise absolute thresholds.

We also conducted a supplementary point of maximum uncertainty (PMU) analysis using trial-level response times. PMU was calculated with a waveform-moment approach, treating the response-time function across SOAs as a waveform and estimating its centre as the response-time-weighted mean SOA for each participant and sequence type (Cacioppo and Dorfman 1987; Bausenhart et al. 2018; Wehrman and Wearden 2023). This estimates the SOA at which responses were slowest, which is interpreted here as a supplementary response-time index of decision uncertainty. Response times shorter than 50 ms or longer than 5000 ms were excluded before PMU calculation; this removed 153 of 12,097 trials. PMU values were analysed with the same mixed ANOVA structure as PSE and JND. Because PMU is response-time based, it is interpreted as a supplementary index of decision uncertainty rather than as a direct measure of subjective alignment.

As an exploratory check, we examined immediate trial-history effects in the trial-level data. The first valid trial in each experimental run was omitted so that previous-trial predictors were defined only within a run. For each participant, linear models predicted either the current late response or the current response time from current SOA, previous-trial SOA, and current sequence type; response-time models used the same 50 ms to 5000 ms inclusion rule as the PMU analysis. In the interleaved group, where same and different previous sequence types were both sufficiently represented, we also tested whether the previous trial had the same sequence type. This check is exploratory because the blocked condition structurally repeated sequence type within runs and therefore did not provide a balanced test of previous sequence type. These analyses were interpreted as checks on carryover rather than as primary tests.

## Results

**Fig. 2:**
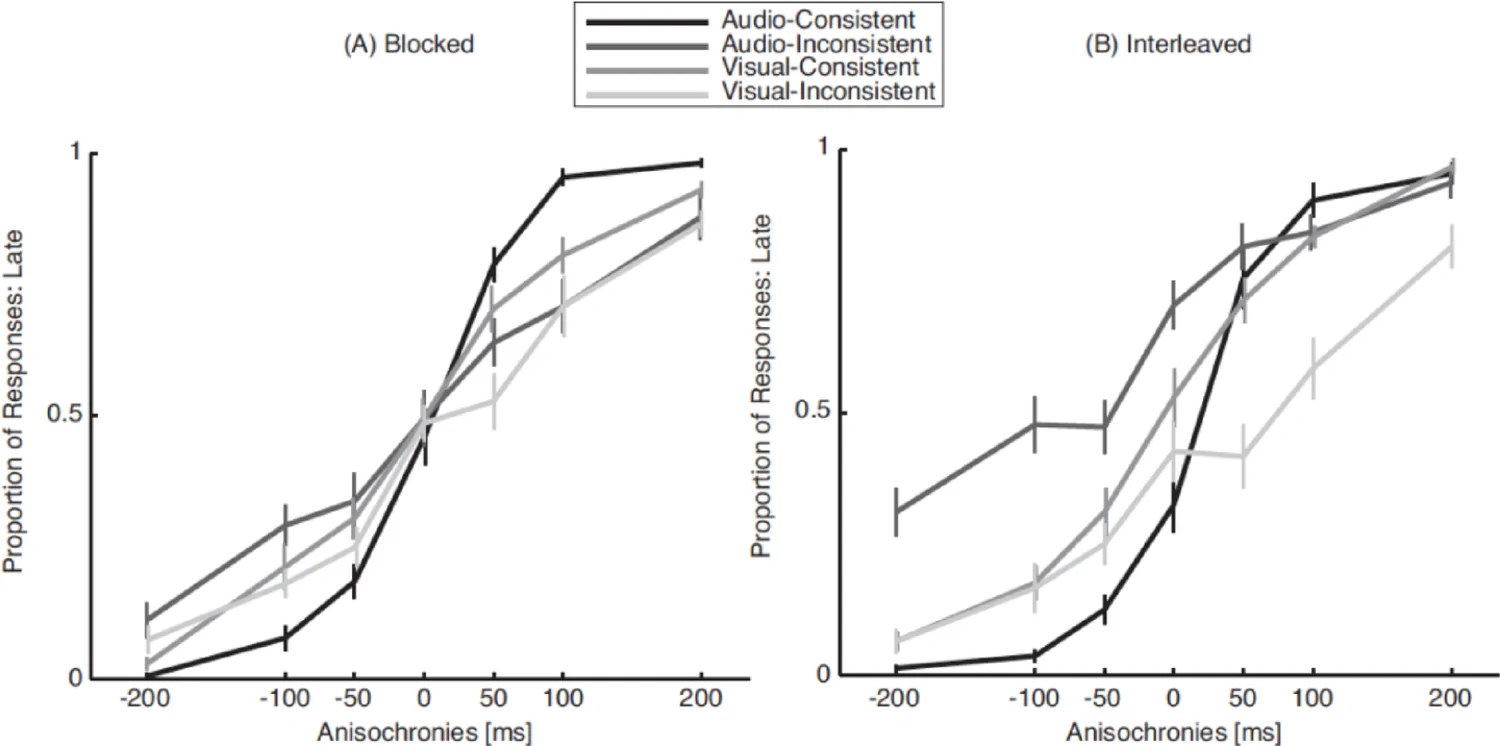
Proportion of late responses as a function of final-event anisochrony, averaged across participants. Analyses were performed on individual data. Panel a shows blocked presentation and panel b shows interleaved presentation. Error bars represent +/− SEM

Figure 2 shows the psychometric functions relating the proportion of late responses to the anisochrony of the final event. Conditions in which the final event matched the modality of the first five events yielded steeper functions than conditions in which the final event switched modality, and this difference was especially evident when the first five events were auditory.

### Temporal sensitivity (JND)

**Fig. 3:**
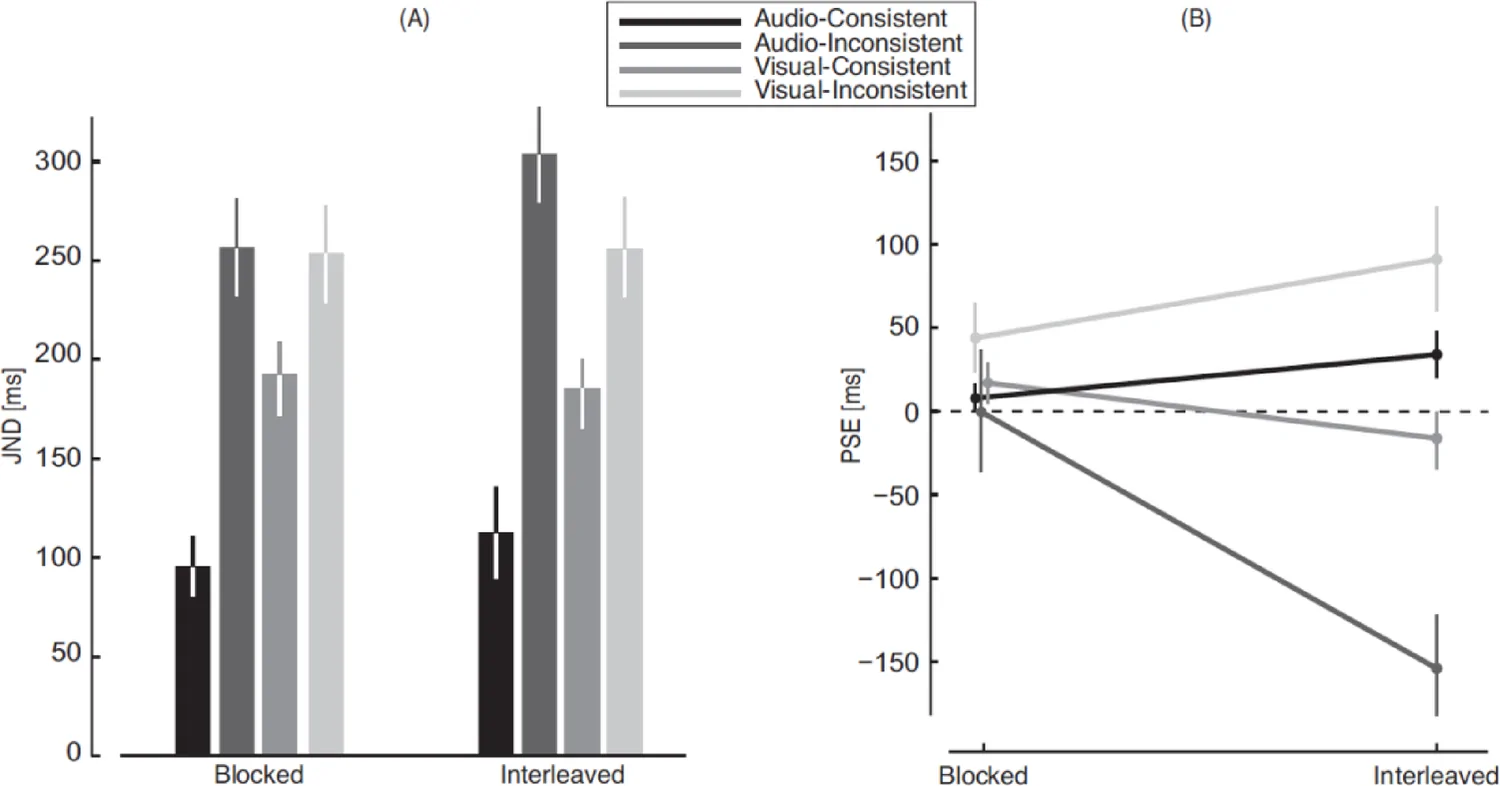
JND and PSE values for the four sequence types as a function of presentation mode. Panel a summarises temporal sensitivity and panel b summarises perceived timing. Error bars represent +/− 1 SEM

JND values are summarised in Fig. 3a. A mixed three-way ANOVA included presentation mode (blocked vs. interleaved) as a between-participants factor and sequence modality (first five events: auditory vs. visual) and consistency (final event: consistent vs. inconsistent) as within-participants factors. Presentation-mode terms are reported descriptively for completeness. There was no reliable main effect of presentation mode, F(1, 34) = 0.46, *p* = 0.503, η_p_^2^ = 0.013. JNDs were lower for auditory than for visual sequences, F(1, 34) = 5.77, *p* = 0.022, η_p_^2^ = 0.145, and lower for modality-consistent than for modality-inconsistent sequences, F(1, 34) = 101.95, *p* < 0.001, η_p_^2^ = 0.750. The presentation mode by modality interaction, F(1, 34) = 2.19, *p* = 0.148, η_p_^2^ = 0.061, and the presentation mode by consistency interaction, F(1, 34) = 0.61, *p* = 0.439, η_p_^2^ = 0.018, were not reliable. Because the modality and consistency effects were qualified by the interaction reported below, interpretation focuses on the Holm-corrected within-participant follow-up tests.

The modality by consistency interaction was significant, F(1, 34) = 20.18, *p* < 0.001, η_p_^2^ = 0.372. The three-way interaction with presentation mode was not reliable, F(1, 34) = 0.12, *p* = 0.731, η_p_^2^ = 0.004. Holm-corrected follow-up tests showed that JNDs were lower for Audio-Consistent than Audio-Inconsistent sequences, mean difference = − 86.6 ms, t(35) = − 9.78, Holm *p* < 0.001, dz = − 1.63, and lower for Visual-Consistent than Visual-Inconsistent sequences, mean difference = − 72.2 ms, t(35) = − 4.34, Holm *p* < 0.001, dz = − 0.72. Auditory sequences were more precise than visual sequences when the final event was consistent, mean difference = − 88.3 ms, t(35) = − 5.07, Holm *p* < 0.001, dz = − 0.85, but not when the final event was inconsistent, mean difference = 26.1 ms, t(35) = 1.38, Holm *p* = 0.177, dz = 0.23.

Thus, temporal sensitivity depended strongly on the modality defining the sequence and on whether the final event maintained or violated that modality pattern. The cost of switching modality was present in both auditory and visual sequences but was larger after auditory sequences. Because some inconsistent-condition JND estimates approached or exceeded the largest absolute anisochrony tested, their absolute magnitudes should be interpreted cautiously.

## Perceived timing (PSE)

PSE values are summarised in Fig. 3b. Negative PSE values indicate that the final event had to occur physically earlier than nominal isochrony to be perceived as regular. Positive PSE values indicate that the final event had to occur physically later than nominal isochrony. The ANOVA on PSE values showed no reliable main effect of presentation mode, F(1, 34) = 3.03, *p* = 0.091, η_p_^2^ = 0.082. There was a main effect of modality, F(1, 34) = 15.92, *p* < 0.001, η_p_^2^ = 0.319, and no reliable main effect of consistency, F(1, 34) = 0.63, *p* = 0.432, η_p_^2^ = 0.018. Presentation-mode terms are reported descriptively because they cannot be separated from the hardware differences between groups. Because the modality effect was qualified by interactions reported below, interpretation focuses on within-group and within-participant simple effects.

PSEs also showed a modality by presentation-mode interaction, F(1, 34) = 5.49, *p* = 0.025, η_p_^2^ = 0.139, a modality by consistency interaction, F(1, 34) = 25.37, *p* < 0.001, η_p_^2^ = 0.427, and a three-way interaction among modality, consistency, and presentation mode, F(1, 34) = 16.39, *p* < 0.001, η_p_^2^ = 0.325. The consistency by presentation-mode interaction was not reliable, F(1, 34) = 2.29, *p* = 0.139, η_p_^2^ = 0.063. Interactions involving presentation mode are therefore used to describe the observed pattern, not to infer a separable causal effect of blocking or interleaving.

The most pronounced follow-up effects were observed within the interleaved group. In this group, Audio-Consistent and Audio-Inconsistent PSEs differed by 187.9 ms, t(17) = 5.81, Holm *p* < 0.001, dz = 1.37, and Audio-Inconsistent and Visual-Inconsistent PSEs differed by − 245.1 ms, t(17) = − 5.31, Holm *p* < 0.001, dz = − 1.25. Because the blocked and interleaved groups also differed in hardware settings, no simple blocked-versus-interleaved follow-up is interpreted as an isolated effect of presentation schedule.

One-sample tests against zero, corrected with Holm’s procedure across the eight sequence-type by presentation-mode tests, are summarised in Table 1. Only the interleaved Audio-Inconsistent condition differed reliably from zero after Holm correction, mean = − 153.9 ms, t(17) = − 5.11, Holm *p* < 0.001, dz = − 1.20. The interleaved Visual-Inconsistent condition showed a positive descriptive shift, mean = 91.2 ms, but this did not survive Holm correction, t(17) = 2.90, Holm *p* = 0.070, dz = 0.68.

Overall, the PSE pattern shows that the largest reliable subjective timing bias occurred for crossmodal sequence endings in the interleaved group. Because presentation mode was between participants and accompanied by hardware differences, this finding should be interpreted descriptively rather than as evidence for a separable blocked-versus-interleaved effect.

The supplementary PMU analysis provided a response-time-based check on decision uncertainty. Mean PMU values were small in absolute magnitude, ranging from − 7.3 ms to 2.8 ms across sequence types and presentation modes. The PMU ANOVA showed a main effect of presentation mode, F(1, 34) = 9.87, *p* = 0.003, η_p_^2^ = 0.225, and a main effect of consistency, F(1, 34) = 6.82, *p* = 0.013, η_p_^2^ = 0.167. The main effect of modality was not reliable, F(1, 34) = 0.00, *p* = 0.957, η_p_^2^ < 0.001. PMU also showed a modality by consistency interaction, F(1, 34) = 35.53, *p* < 0.001, η_p_^2^ = 0.511, and a modality by consistency by presentation-mode interaction, F(1, 34) = 8.46, *p* = 0.006, η_p_^2^ = 0.199. As for PSE and JND, presentation-mode terms in the PMU analysis are descriptive because they are confounded with hardware settings.

Holm-corrected PMU follow-up tests were interpreted as within-group sequence-type and modality contrasts. In the interleaved group, PMU differed between Audio-Consistent and Audio-Inconsistent sequences, mean difference = 10.1 ms, t(17) = 9.00, Holm *p* < 0.001, dz = 2.12, and between Visual-Consistent and Visual-Inconsistent sequences, mean difference = − 5.4 ms, t(17) = − 3.13, Holm *p* = 0.031, dz = − 0.74. PMU also differed between modalities for consistent sequences, mean difference = 6.7 ms, t(17) = 5.45, Holm *p* < 0.001, dz = 1.28, and inconsistent sequences, mean difference = − 8.8 ms, t(17) = − 5.17, Holm *p* < 0.001, dz = − 1.22. In the blocked group, only the modality effect for consistent sequences survived Holm correction, mean difference = 3.8 ms, t(17) = 2.98, Holm *p* = 0.033, dz = 0.70. Thus, PMU was sensitive to the same broad modality-consistency structure as PSE, but the absolute PMU shifts were much smaller.

The exploratory trial-history analysis did not provide evidence that the main PSE context effect was explained by simple carryover from the immediately preceding trial. Previous SOA showed a small negative association with current late responses in the blocked group, − 1.23% points per + 100 ms previous SOA, t(17) = − 2.37, *p* = 0.030, but not in the interleaved group, − 0.24% points, t(17) = − 0.46, *p* = 0.651. Because sequence history differed structurally between presentation modes, these slopes were not interpreted as a formal blocked-versus-interleaved comparison. Previous SOA did not reliably predict response times. In the interleaved group, same versus different previous sequence type did not predict current late responses, − 0.47% points, t(17) = − 0.44, *p* = 0.667, although same-type repeats were associated with shorter response times, − 82.6 ms, t(17) = − 3.54, *p* = 0.003.

**Table 1:** Holm-corrected one-sample tests of PSE values against zero

Presentation mode	Test	Audio-consistent	Audio-inconsistent	Visual-consistent	Visual-inconsistent
Blocked	t(17)	0.96	0.11	1.37	2.16
	Holm p	0.974	0.974	0.757	0.227
Interleaved	t(17)	2.45	− 5.11	− 1.01	2.90
	Holm p	0.153	< 0.001	0.974	0.070

Holm-corrected* p* values are shown for tests of PSE values against zero within each sequence type and presentation mode. Only the interleaved Audio-Inconsistent condition remained significant after correction

## Discussion

The present study examined how sequence-level modality history and across-trial modality context contribute to isochrony judgments. We distinguish this question from classical temporal recalibration, because the present design did not expose participants to repeated bimodal stimulus pairs with a fixed temporal offset. Instead, it tested whether the precision and subjective alignment of a final event depend on the modality pattern established within a sequence and on whether sequence types are blocked or interleaved across trials.

The JND results extend previous evidence that auditory timing is more precise than visual timing and show that this advantage persists when the judgment concerns the final event in a regular sequence (Repp and Penel 2002; Stauffer et al. 2012; Fornaciai et al. 2018). More importantly, sensitivity decreased when the modality of the final event differed from the modality that had established the sequence. This switch cost was larger after auditory sequences than after visual sequences. One parsimonious interpretation is that auditory sequences established a relatively precise temporal template, so replacing the expected auditory final event with a visual event imposed a larger cost. Visual sequences were already less precise, leaving less additional loss of sensitivity when the final event switched modality.

This result is compatible with the idea that the temporal comparison underlying JND was constrained by modality-specific sequence history. It does not require a fully modality-independent temporal template. The finding is also consistent with theories in which temporal expectation is tied to contextual features of prior events. For example, Multiple Trace Theory of Temporal Preparation proposes that temporal regularities can be stored together with non-temporal event features in memory traces (Los et al. 2014). Although the present study was not designed as a direct test of that theory, the observed modality-switch cost is consistent with the broader proposal that temporal expectation can include stimulus-feature information.

By contrast, we did not obtain reliable evidence that the presentation schedule altered JND in this sample. This should not be interpreted as evidence that contextual modality knowledge can never affect temporal sensitivity. In the present experiment, all deviations occurred at the same temporal position, namely the final event. Blocking therefore provided knowledge about the likely modality pattern across trials, but not about when a deviation might occur. Under those conditions, modality-specific sequence history may be the dominant determinant of precision. The fixed SOA range also means that very large JND estimates, especially in inconsistent conditions, should be treated as relative indices of reduced sensitivity rather than exact threshold values.

The PSE results point to a different level of temporal processing. In the interleaved group, crossmodal sequences showed large and opposite shifts depending on the modality of the final event: a final visual event embedded in an auditory sequence was associated with a negative PSE, whereas a final auditory event embedded in a visual sequence was associated with a positive PSE. The blocked group did not show the same reliable one-sample deviations from zero, but this between-group pattern cannot be attributed uniquely to presentation schedule because hardware settings also differed. The strongest inference supported by the present data is therefore descriptive: subjective alignment biases were large for crossmodal sequence endings under interleaved presentation. The data do not determine whether this pattern reflects a change in perceptual latency, a change in decisional reference, response strategy, or a combination of these factors.

The direction of the PSE effects can be understood in terms of final-event modality switches. In the interleaved group, a visual final event after an auditory sequence had to occur physically earlier to be judged as isochronous, whereas an auditory final event after a visual sequence tended to require the opposite adjustment. This directional pattern is consistent with the idea that observers assigned different subjective timing to auditory and visual final events when the modality context was uncertain. It is also compatible with prediction-violation accounts, in which an unexpected final-event modality changes the interpretation of the event’s timing. However, the present data do not isolate a predictive-coding mechanism, and this account should therefore be treated as a possible interpretation rather than a demonstrated process.

The dissociation between JND and PSE is theoretically informative because it constrains what can be concluded about temporal expectation. A single undifferentiated account would predict that the same contextual information should shift both precision and bias in parallel. In this sample, the strongest evidence for precision effects concerned modality-specific sequence history, whereas the clearest bias was observed for crossmodal events in the interleaved group. The data are therefore more consistent with a partially separable architecture than with a single undifferentiated timing mechanism.

The supplementary PMU analysis provides converging but more limited evidence. PMU was affected by modality and consistency in within-group contrasts, indicating that response times were slowest at different SOAs depending on the sequence context. This is consistent with the idea that participants’ uncertainty was also context dependent. However, the PMU shifts were only a few milliseconds, much smaller than the PSE shifts, and should therefore be treated as a supplementary decision-time measure rather than as evidence for large changes in perceived event timing.

The exploratory trial-history analysis further qualifies the context interpretation. The analysis did not show that previous sequence type predicted the current early-late judgement in the interleaved group, although same-type repeats were associated with faster responses. This pattern is more consistent with response or decision facilitation than with a shift in subjective alignment. A small previous-SOA association with late responses appeared in the blocked group, but this exploratory effect was not taken as a presentation-mode comparison. Thus, the available trial-level data do not indicate that the main PSE context effect is explained by simple carryover from the preceding SOA or sequence type.

Several limitations should be addressed in future work. First, presentation mode was manipulated between participants, and the blocked and interleaved groups were tested with different stimulus devices and intensity settings. These differences do not explain the strong within-participant effects of modality and consistency, but they limit any causal inference about blocked versus interleaved presentation. Second, the current design cannot distinguish whether the critical noise in inconsistent trials should be attributed to the final event’s modality, to the crossmodal interval type itself (A-V, auditory then visual, vs. V-A, visual then auditory), or to both. A more decisive design would include additional alternating sequences that permit separate estimation of A-V and V-A interval precision. Third, the PMU and sequential-effect analyses were exploratory and used simple linear summaries of the trial-level data. Future work should pre-register any decision-time or trial-history analyses and include conditions designed specifically to test PMU and sequential-dependency predictions. Finally, extending the design to other modality pairs would help determine whether the contextual alignment effects observed here reflect a general multisensory principle or a stronger specialisation of audiovisual timing.

In summary, temporal sensitivity in regular sequences was shaped mainly by the modality pattern established within a trial, whereas perceived timing showed the clearest bias for crossmodal endings in the interleaved condition. Crossmodal sequence endings therefore reveal a dissociation between precision and bias. The broader implication is that temporal judgment in multisensory sequences should not be treated as the unitary readout of one mechanism, but as reflecting separable contributions to temporal precision and subjective alignment.

## Data Availability

The data and analysis scripts that support the findings of this study are available from https://github.com/maxdiluca/unimodal-crossmodal-sequences.
